# Covalent immobilization of β-galactosidase using a novel carrier alginate/tea waste: statistical optimization of beads modification and reusability

**DOI:** 10.1007/s00449-023-02959-1

**Published:** 2024-01-10

**Authors:** Mohamed A. A. Abdella, Mohamed E. Hassan

**Affiliations:** 1 Chemistry of Natural and Microbial Products Department, Pharmaceutical and Drug Industries Research Institute, National Research Centre, Dokki, Giza 12622 Egypt; 2 Centre of Excellence, Encapsulation and Nanobiotechnology Group, National Research Centre, Dokki, Giza 12622 Egypt

**Keywords:** Immobilized β-galactosidase, Alginate/tea waste, Chemical modification, Statistical optimization, Reusability

## Abstract

β-galactosidase has been immobilized onto novel alginate/tea waste gel beads (Alg/TW) via covalent binding. Alg/TW beads were subjected to chemical modification through amination with polyethyleneimine (PEI) followed by activation with glutaraldehyde (GA). Chemical modification parameters including PEI concentration, PEI pH, and GA concentration were statistically optimized using Response Surface methodology (RSM) based on Box–Behnken Design (BBD). Analysis of variance (ANOVA) results confirmed the great significance of the model that had *F* value of 37.26 and *P* value < 0.05. Furthermore, the *R*^2^ value (0.9882), Adjusted *R*^2^ value (0.9617), and predicted *R*^2^ value (0.8130) referred to the high correlation between predicted and experimental values, demonstrating the fitness of the model. In addition, the coefficient of variation (CV) value was 2.90 that pointed to the accuracy of the experiments. The highest immobilization yield (IY) of β-galactosidase (75.1%) was given under optimized conditions of PEI concentration (4%), PEI pH (9.5), and GA concentration (2.5%). Alg/TW beads were characterized by FT-IR, TGA, and SEM techniques at each step of immobilization process. Moreover, the immobilized β-galactosidase revealed a very good reusability as it could be reused for 15 and 20 consecutive cycles keeping 99.7 and 72.1% of its initial activity, respectively. In conclusion, the environmental waste (tea waste) can be used in modern technological industries such as the food and pharmaceutical industry.

## Introduction

Tea is the most popular and widely used non-alcoholic beverage in the world. Using tea alone is equivalent to consuming chocolate, soft drinks, alcohol, and coffee all together [1]. Due to its numerous health advantages and numerous reviving effects, tea use is exponentially growing around the world [2]. The value of the global tea market was estimated at 200 billion USD in 2020 and is expected to reach over 318 billion USD by the year 2025 [3].

Black tea contains structural proteins (soluble and insoluble), polysaccharides that dissolve in hot water [4]. Essentially, tea contains a variety of biologically active ingredients including methylxanthines, alkaloids (caffeine, theophylline, and theobromine), polyphenols (catechins, flavonoids, and proanthocyanidins), pigments, terpenoids, vitamins, amino acids, and polysaccharides [5–7]. Tea waste frequently contains nearly identical amounts of components as regular tea [8].

Waste from tea production amounts to billions of tons practically everywhere in the world, costing money and having negative effects on the environment for disposal [9]. These wastes are burned or disposed of in landfills. To change customer behavior and reduce waste, the tea industry is currently playing a significant role in the adoption of new technology. In addition to looking into new inventions and sustainable practices [10, 11].

Tea waste is widely utilized in a variety of businesses, including those that deal with the environment, energy, polymer manufacturing, electrical appliances, etc. Due to its low cost, abundant supply, eco-friendliness, and renewability, which are the main factors taken into account for sustainable development tea is a material that shows promise for a variety of uses [3].

Alginate, chitosan, chitin, starch, and their derivatives are examples of biomaterials that have undergone substantial study because of their abundance of resources, biocompatibility, lack of toxicity, and ease of modification due to the wide variety of functional groups on their surfaces. Alginate is one of the best materials for enzyme immobilization technologies via entrapment or covalent techniques. Alginate and tea waste together offer numerous benefits throughout the immobilization process [12].

The carrier was prepared by improving the mechanical properties of alginate by adding tea waste, which raises the active groups on the surface of the alginate/tea waste complex. The complex can then be used in the immobilization process by forming covalent bonds with the enzyme. The covalent bonds can be obtained by treating the carrier with PEI and GA. This polymer complex can be used several times for enzyme immobilization, which reduces environmental waste and the total cost of the products. This new carrier eliminated this problem, and the results showed the high activity of the enzyme, making it more applicable in medicine and industry.

The enzyme β-galactosidase (β-gal (EC 3.2.1.23)) can hydrolyze the bonds in oligo- and disaccharides. It can be obtained from animals, plants, and microbial sources such as bacteria, fungi, and yeasts. Lactose is the main substrate for this enzyme. The enzymatic hydrolysis of lactose by β-gal has two main biotechnological applications: the production of lactose-free milk and dairy products for consumption by lactose intolerant persons and the utilization of whey, as its hydrolysates (glucose and galactose) have a higher fermentation potential [13]. So that β-gal was selected as a model enzyme for the immobilization study.

The conventional way for optimizing a multivariable system by one-variable-at-a-time (OVAT) method is not only time consuming but also expensive, laborious and unsuccessful to examine the interactions between different variables [14]. So, optimization using statistical design experiments can be performed to overcome this problem [15]. Response surface methodology (RSM) is a collection of statistical and mathematical techniques used for designing experiments and determining the optimal conditions influencing the response (IY %) based on the fit of a polynomial equation to the experimental data [16]. Moreover, Box–Behnken design (BBD) of RSM is a three-level factorial model used to decrease the number of trails required for testing the interactions between different variables and identifying the most significant ones [17, 18].

In the present study, β-gal enzyme was immobilized onto a novel alginate/tea waste (Alg–TW) carrier using covalent binding method. Chemical modification parameters of Alg/TW beads were optimized using a statistical method to enhance the IY%. FT-IR, TGA, and SEM techniques were used to examine the carrier preparation and enzyme immobilization steps. Finally, the reusability of immobilized β-gal was examined for several operational cycles and its residual activity (Res A %) was determined.

## Materials and methods

### Materials

Sodium alginate was purchased from Fluka. Polyethyleneimine (PEI), Glutaraldehyde solution (GA), and the *Aspergillus oryzae* β-D-gal (EC 3.2.1.23) were purchased from Sigma-Aldrich, Germany. Tea waste was collected from the famous tea brand in Egypt. Other chemicals were of Analar or equivalent quality.

### Methods

#### Tea waste preparation

The tea waste was prepared by boiling the tea well to remove impurities and dyes, after which the tea waste was left to dry at room temperature. It was finely ground and a sieve (sized to a particle size of 500 μm to 1 mm) was used to obtain relatively small and consistent particles.

#### Preparation of alginate/tea waste gel beads

**Fig. 1:**
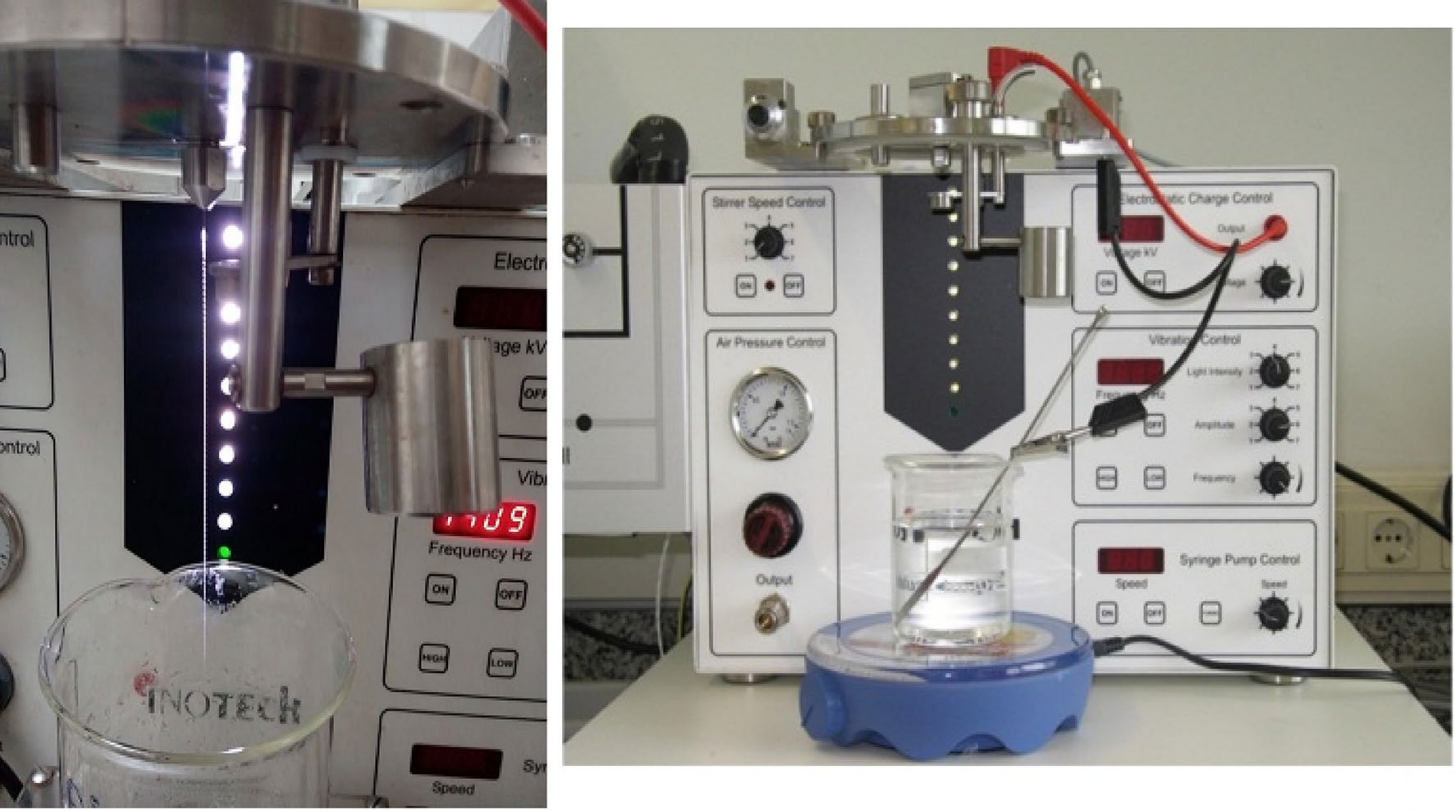
Encapsulator for making uniform gel beads

A homogeneous mixture of alginate and tea waste was prepared in a ratio of 1:1 with a final concentration of 2%. Then, using a 300 mm nozzle of the encapsulator shown in Fig. 1, the mixture was dropped into a 3% calcium chloride solution and the gel granules were left for 3 h to solidify completely.

### Statistical optimization of beads modifications

#### Box–Behnken design

Box–Behnken design (BBD) of Response Surface methodology (RSM) was implemented to optimize the most important variables affecting beads modification in covalent binding of Alg/TW carrier for highest immobilization yield (IY) of β-gal [19]. In this study, the investigation of three independent variables (PEI concentration, PEI pH and GA concentration) was carried out at low (– 1), central (0) and high (+ 1) levels. Based on BBD, 14 experimental trials were resulted from the combinations between the selected variables with respect to mean of IY (%) as response. The data of BBD were demonstrated by a quadratic model using the following equation:

$${\text{Y}} = \beta_{0} + \Sigma \beta_{{\text{i}}} {\text{X}}_{{\text{i}}} + \Sigma \beta_{{{\text{ii}}}} {\text{X}}_{{\text{i}}}^{{2}} + \Sigma \beta_{{{\text{ij}}}} {\text{X}}_{{\text{i}}} {\text{X}}_{{\text{j}}}$$

where: *Y*, the predicted response; *β*_0_, the intercept term; *β*_*i*_, the linear coefficient; *β*_*ii*_, the quadratic coefficient; *β*_*ij*_, the interaction coefficient; and *X*_*i*_, *X*_*j*_, the independent variables.

#### Data analysis and software

The significance of the regression model and each term in equation was determined by Analysis of variance (ANOVA) based on *F* test and probability value (*P* value). Moreover, the quality of fit for the statistical model equation was evaluated by the determination coefficient (*R*^2^) and the Adjusted *R*^2^ [20]. Design Expert 13.0 (trial version, Stat Ease Inc., Minneapolis, MN, USA) statistical software was used for experimental design, analyzing and interpreting the experimental data.

#### Activation of Alg/TW gel beads

**Scheme 1:**
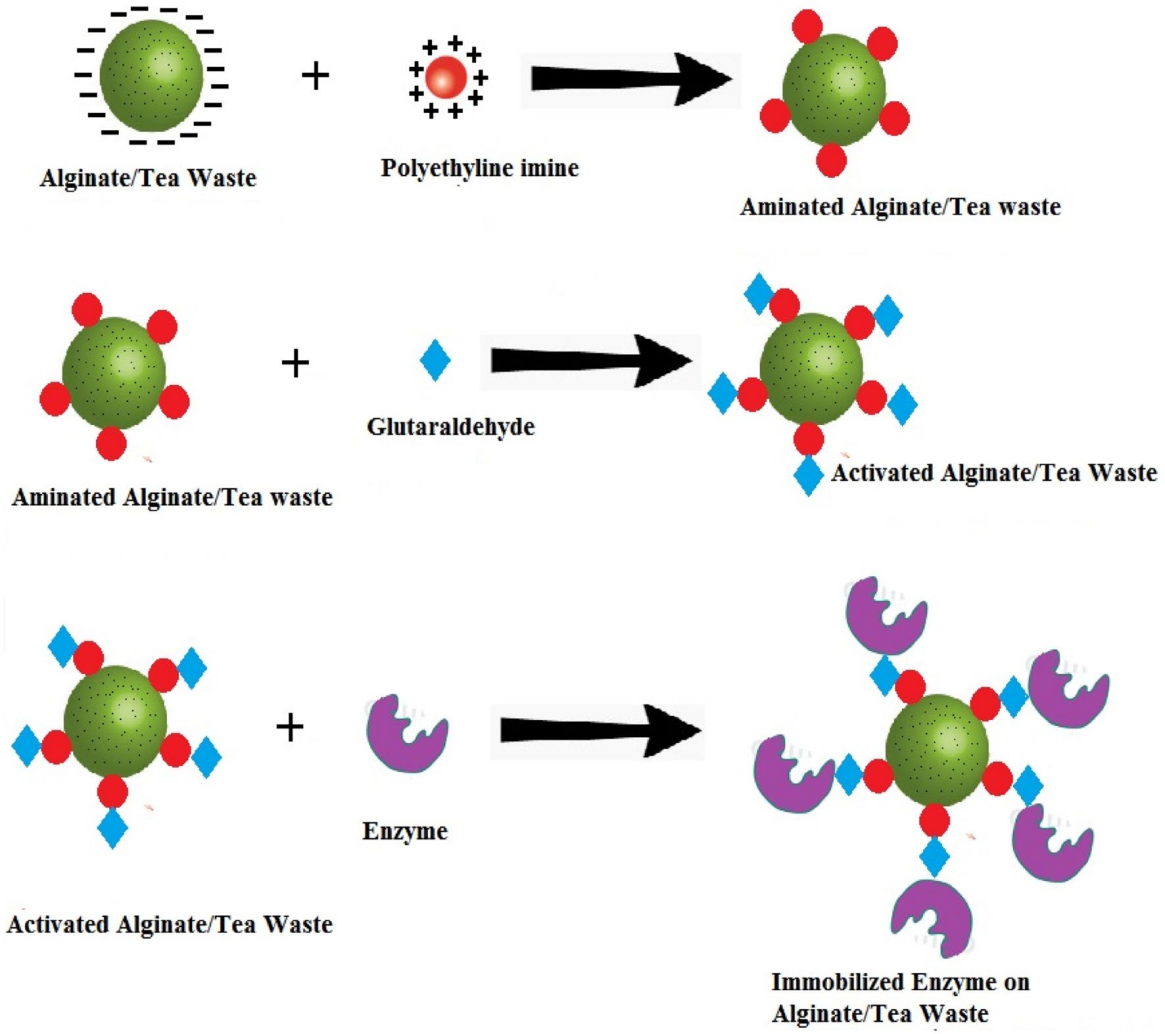
Activation of alginate/tea waste complex and immobilization processes

Polyethyleneimine (PEI) and glutaraldehyde (GA) were applied to activate Alg/TW gel beads for covalent immobilization. The gel beads were thoroughly rinsed three times to get rid of any PEI that had not fully reacted after being steeped in a 4% PEI solution at 9.5 pH for three h. The aminated gel beads were then submerged in a 2.5% GA solution for three h. The gel beads were thoroughly washed with distilled water to eliminate the unreacted GA as in Scheme 1. The activated gel beads were now prepared for additional immobilization procedures [21].

#### β-galactosidase assay

The activity of β-gal can be estimated by liberating O-nitrophenol (ONP) from O-nitrophenyl-β-D-galactopyranoside (ONPG) as a substrate according to the method characterized by Zhou et al. [22] with some modifications. The reaction was started by adding 0.1 ml of the enzyme solution (in the case of a free enzyme) or 0.1 g of beads (in the case of an immobilized enzyme) to 0.9 ml of 10 mM ONPG solution prepared in phosphate buffer (0.1 M, pH 7.0). Then, the reaction mixture was incubated at 40 °C for 15 min and stopped by adding 0.5 ml of Na_2_CO_3_ (1 M). The released ONP was determined by measuring the absorbance at 420 nm. One unit (U) of β-gal activity was defined as the amount of enzyme required to release 1 µmole of ONP per minute under the assay conditions [23].

#### β-galactosidase immobilization

β-galactosidase was attached to the activated Alg/TW carrier via covalent immobilization. The binding was carried out by mixing 2.0 ml of β-gal (600 U) with 1 g of activated Alg/TW beads, and then the mixture was left overnight at 4 °C. After that, the beads were washed twice with distilled H_2_O and used for activity assay of immobilized β-gal. The immobilization yield (IY %) was calculated according to the following equation [12, 24].

$${\text{IY}}\left( \% \right) = \left( {\text{I/A}} \right) \times {1}00$$

where: *I*, the activity of immobilized enzyme and *A*, the total activity offered for immobilization.

### Characterization of the prepared Alg/TW gel beads

#### Fourier transform infrared spectroscopy (FT-IR)

To find out the modifications of functional groups on the surface of the four formulas (Alg/TW, Alg/TW/PEI, Alg/TW/PEI/GA, and Alg/TW/PEI/GA/Enz.). Reduced total reflection Fourier transform infrared spectroscopy (ATR-FT-IR) was used to identify the main function groups. ATR-FT-IR analysis of the gel beads was conducted using a FT-IR spectrophotometer (Shimadzu FT-IR-800, Japan). Examined samples were scanned in the range of 4000 to 400 cm^−1^.

#### Thermal gravimetric analysis (TGA)

TGA was performed to prove the formation of a strong polyelectrolyte complex. The thermal behavior of the different formulations (Alg/TW, Alg/TW/PEI, Alg/TW/PEI/GA, and Alg/TW/PEI/GA/Enz.) was characterized by the TGA (Themys one+, USA).

#### Scanning electron microscopy (SEM)

Scanning electron microscopy (FEI Quanta 3D 200i Edx / thermo fisher pathfinder operated under conditions of high vacuum for acceleration voltage 5.0 ~ 10.0 kv using secondary electron detector with working distance 15–17 mm) was used to image the fracture surfaces of the four formulas (Alg/TW, Alg/TW/PEI, Alg/TW/PEI/GA, and Alg/TW/PEI/GA/Enz.).

#### Operational stability (reusability) of immobilized β-galactosidase

The reusability of immobilized β-gal was investigated up to 20 cycles by the addition of 1 ml of the substrate (ONPG/0.1 M phosphate buffer, pH 7.0) to 0.1 g beads of immobilized β-gal and incubation in water bath at 40 °C for 15 min. The beads were washed after each cycle with 0.1 M phosphate buffer (pH 7.0) to remove the substrate residues and will be reused to initiate a new reaction cycle. The activity of immobilized enzyme in the 1st cycle was taken as 100% and the residual activity (Res *A* %) was determined using the following formula as suggested by [25]

$${\text{Res }}A\% = \left( {\text{Obtained activity/Initial activity}} \right) \times {1}00$$

## Results and discussion

### BBD for optimizing beads modifications

BBD as displayed in Table 1 was carried out to get the optimum levels of the tested variables required for beads modification in covalent immobilization. According to the statistical analysis of BBD results, a second-order polynomial model was generated which fitted to the following equation:

$$Y = + 74.85 + 4.29A - 1.16B - 1.15C - 3.57AB - 1.50AC + 2.85BC - 9.49A^{2} - 9.24B^{2} - 11.01C^{2}$$

where: *Y* is the predicted response (IY %); *A*, *B*, and *C* are the code values of PEI concentration, PEI pH, and GA concentration, respectively.

**Table 1:** BBD for optimizing beads modifications affecting β-galactosidase immobilization

Trial	A:PEI concentration	B:PEI pH	C:GA concentration	Immobilization yield	Predicted values
	%	–	%	%	%
1	(– 1) 2	(– 1) 8.5	(0) 2.5	50	49.43
2	(0) 4	(0) 9.5	(0) 2.5	75.1	74.85
3	(0) 4	(0) 9.5	(0) 2.5	75.1	74.85
4	(0) 4	(+ 1) 10.5	(– 1) 1	52.2	51.74
5	(+ 1) 6	(+ 1) 10.5	(0) 2.5	55.1	55.68
6	(+ 1) 6	(0) 9.5	(– 1) 1	61.4	61.29
7	(– 1) 2	(0) 9.5	(– 1) 1	48.1	49.71
8	(0) 4	(– 1) 8.5	(– 1) 1	60.8	59.76
9	(– 1) 2	(+ 1) 10.5	(0) 2.5	55.4	54.25
10	(+ 1) 6	(0) 9.5	(+ 1) 4	57.6	55.99
11	(0) 4	(– 1) 8.5	(+ 1) 4	51.3	51.76
12	(+ 1) 6	(– 1) 8.5	(0) 2.5	64	65.15
13	(– 1) 2	(0) 9.5	(+ 1) 4	50.3	50.41
14	(0) 4	(+ 1) 10.5	(+ 1) 4	54.1	55.14

ANOVA for BBD was used to determine if the model and its tested variables were significant. As illustrated in Table 2, the model *F* value was 37.26 and *P* value < 0.05 that indicate the model was significant. There is only a 0.17% chance that, such a large model *F* value could occur due to noise. The data points to the great difference in response which can be proved by the model equation. On the other hand, the determination coefficient (*R*^2^) value is considered a significant tool to demonstrate the efficiency of the statistical model. In our case, the *R*^2^ value (0.9882) and Adjusted *R*^2^ value (0.9617) implied that the regression model was effective and revealed a good explanation of the relationship between the independent variables and IY of β-gal. In addition, the good agreement of predicted *R*^2^ value (0.8130) provided a great correlation between the observed and theoretical values predicted by the statistical model. Furthermore, the coefficient of variation (CV) points to the degree of accuracy and reliability of the experimental results [19, 26]. The low CV value (2.90) refers to the good performance of the experiments. Moreover, the lack of fit was non-significant (*P* value = 0.1340) which proved that the model equation was fitted to the predict response (IY) [27].

**Table 2:** ANOVA of BBD for optimizing beads modifications

Source	Sum of squares	df	Mean Square	Std. Dev	*F* value	*P* value	
Model	942.22	9	104.69	1.68	37.26	0.0017	Significant
A-PEI concentration	147.06	1	147.06	1.57	52.35	0.0019	
B-PEI pH	10.81	1	10.81	0.7845	3.85	0.1213	
C-GA concentration	10.58	1	10.58	1.18	3.77	0.1243	
*AB*	51.12	1	51.12		18.20	0.0130	
*AC*	9.00	1	9.00		3.20	0.1480	
*BC*	32.49	1	32.49		11.56	0.0273	
*A*^*2*^	288.04	1	288.04		102.53	0.0005	
*B*^*2*^	273.06	1	273.06		97.20	0.0006	
*C*^*2*^	388.08	1	388.08		138.14	0.0003	
Residual	11.24	4	2.81				
Lack of Fit	11.11	3	3.70		29.63	0.1340	Non-significant
Pure Error	0.1250	1	0.1250				
Cor Total	953.45	13					

*R*^2^ = 0.9882, Adjusted *R*^2^ = 0.9617, Predicted *R*^2^ = 0.8130, CV = 2.90%, Adequate Precision = 17.9482

*Std. Dev.* standard deviation, *df* degree of freedom, *Significant* *P* < 0.05, *Non-significant* *P* > 0.05

Moreover, Table 3 displayed the regression coefficients, standard errors and *P* values of linear (*A*, *B*, *C*), squared (*A*^2^, *B*^2^, *C*^2^) and interaction (*AB*, *AC*, *BC*) model terms. The results indicated that, linear term (*A*), interaction terms (*AB*, *BC*) and squared terms (*A*^2^, *B*^2^, *C*^2^) were significant (*P* value < 0.05) while linear terms (*B*, *C*) and interaction term (*AC*) were non-significant (*P* value > 0.05).

**Table 3:** Coefficients, standard errors and *P* values of BBD for optimizing beads modifications

Term	Coefficient estimate	Standard error	*P* value	95% CI Low	95% CI High	VIF
Intercept	74.85	1.19		71.56	78.14	
A-PEI concentration	4.29	0.5926	0.0019	2.64	5.93	1.0000
B-PEI pH	– 1.16	0.5926	0.1213	– 2.81	0.4828	1.0000
C-GA concentration	– 1.15	0.5926	0.1243	– 2.80	0.4953	1.0000
*AB*	– 3.57	0.8381	0.0130	– 5.90	– 1.25	1.0000
*AC*	– 1.50	0.8381	0.1480	– 3.83	0.8268	1.0000
*BC*	2.85	0.8381	0.0273	0.5232	5.18	1.0000
*A*^*2*^	– 9.49	0.9370	0.0005	– 12.09	– 6.89	1.07
*B*^*2*^	– 9.24	0.9370	0.0006	– 11.84	– 6.64	1.07
*C*^*2*^	– 11.01	0.9370	0.0003	– 13.61	– 8.41	1.07

**Fig. 2:**
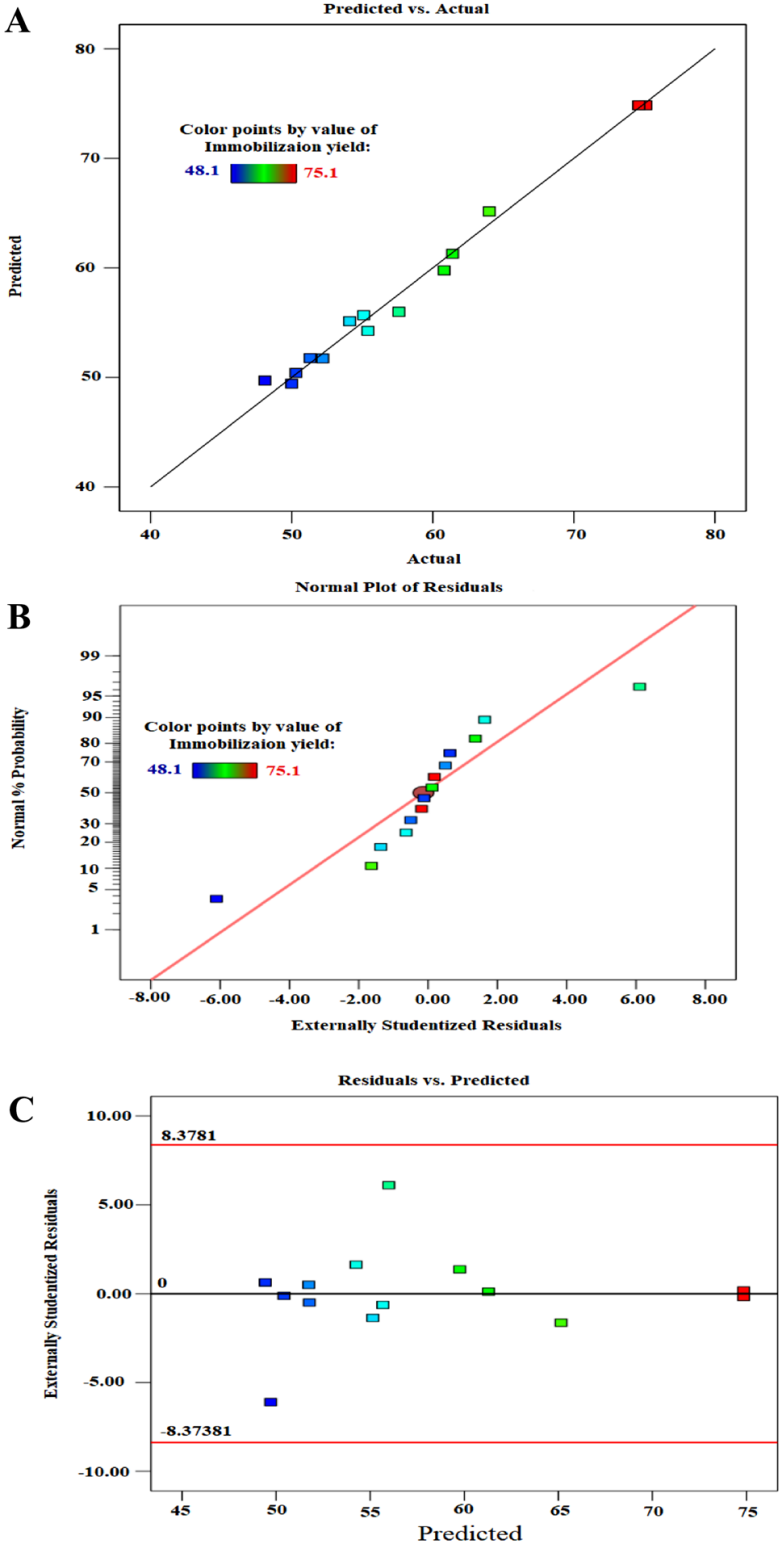
**A** The relation between predicted and actual values **B** Normal probability plot of residuals **C** Plot of residuals vs. predicted values of β-galactosidase IY% using BBD for optimizing beads modifications

The statistical model validation was achieved by comparing the predicted IY with the actual IY values. As seen in Fig. 2A, there was high degree of accordance between predicted and experimental values that demonstrates the validity and fitness of the model. In addition, the normal probability plot of residuals shows the closing of the residual points along a straight line that implies the model was adequate to the experimental results (Fig. 2B). On the other side, the plot of residuals versus the predicted response values exhibits the scattering of residuals randomly around a horizontal zero reference which points to the effectiveness of the regression model as shown in Fig. 2C.

**Fig. 3:**
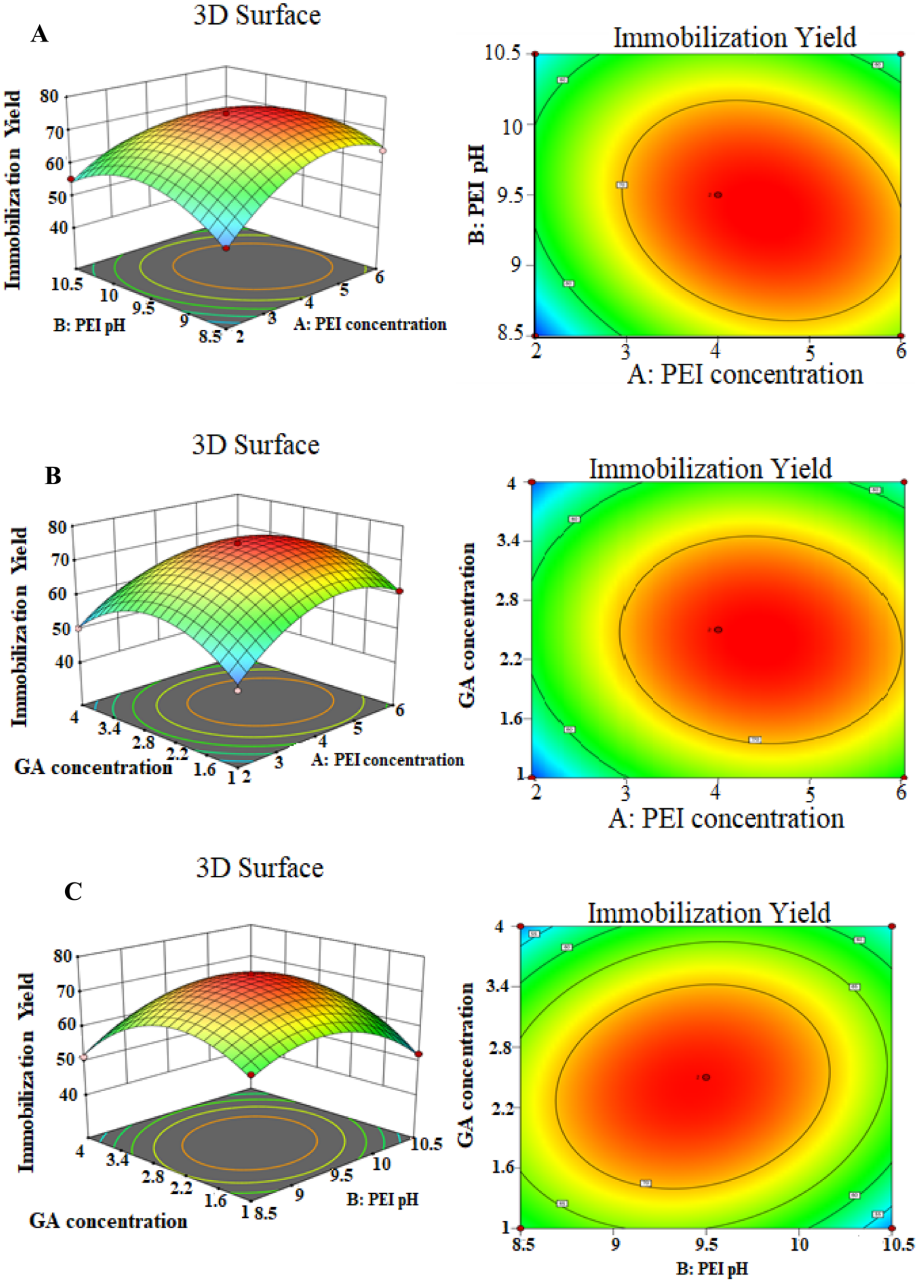
The 3D response surface and contour plots showing interactions between each two variables affecting beads modifications **A** PEI concentration and PEI pH **B** PEI concentration and GA concentration **C** PEI pH and GA concentration

The 3D response surface and contour plots of BBD showed the interactions between two different variables while keeping the other one at its central level (Fig. 3). These graphs give a visual interpretation for the effect of interactions among the examined variables on IY to determine their optimum levels. Figure 3A displays the interaction between PEI concentration and PEI pH keeping GA concentration at its zero level (2.5%). The highest IY (75.1%) takes place at the central levels of both PEI concentration (4%) and PEI pH (9.5). On the other hand, Fig. 3B shows the effect of PEI concentration and GA concentration on the IY, whereas PEI pH was kept at its zero level (9.5). The highest IY (75.1%) occurs at the central levels of both PEI concentration (4%) and GA concentration (2.5%). Furthermore, Fig. 3C illustrates the interaction between PEI pH and GA concentration preserving PEI concentration at its central level (4%). The maximum IY (75.1%) was obtained at zero level of PEI pH (9.5) and zero level of GA concentration (2.5%).

Finally, the highest IY of β-galactosidase (75.1%) was observed in trial 2 under optimizing conditions of PEI concentration (4%), PEI pH (9.5), and GA concentration (2.5%). Other studies on optimizing beads modification for enzyme immobilization reported that, the maximum IY (81.6%) was obtained at 6% PEI and 4% GA [16]. In addition, the highest inulinase IY (60%) on GEG-Alg-SPI carrier was achieved at PEI concentration (1.5%), PEI pH (7.0), and (GA) concentration (5%) [28].

The immobilization efficiency (75.66%) of β-gal onto Sephadex G-75 beads was achieved using 4.4% GA at pH 7, whereas it was 74.92% when β-gal was immobilized onto chitosan beads at 1.3% GA, pH 6, as suggested by [29]. In covalent immobilization of β-gal on gellan gum (GG) beads, the polyamine treatment step was optimized using a central composite design, and it was found that the best values for PEI concentration and PEI pH were 6.15% and 8.30, respectively [30]. Further, RMS based on BBD was used to determine the optimal immobilization conditions of β-gal onto graphene nano-sheets, which resulted in 84.2% immobilization efficiency [31].

### Fourier transform infrared spectroscopy (FT-IR)

**Fig. 4:**
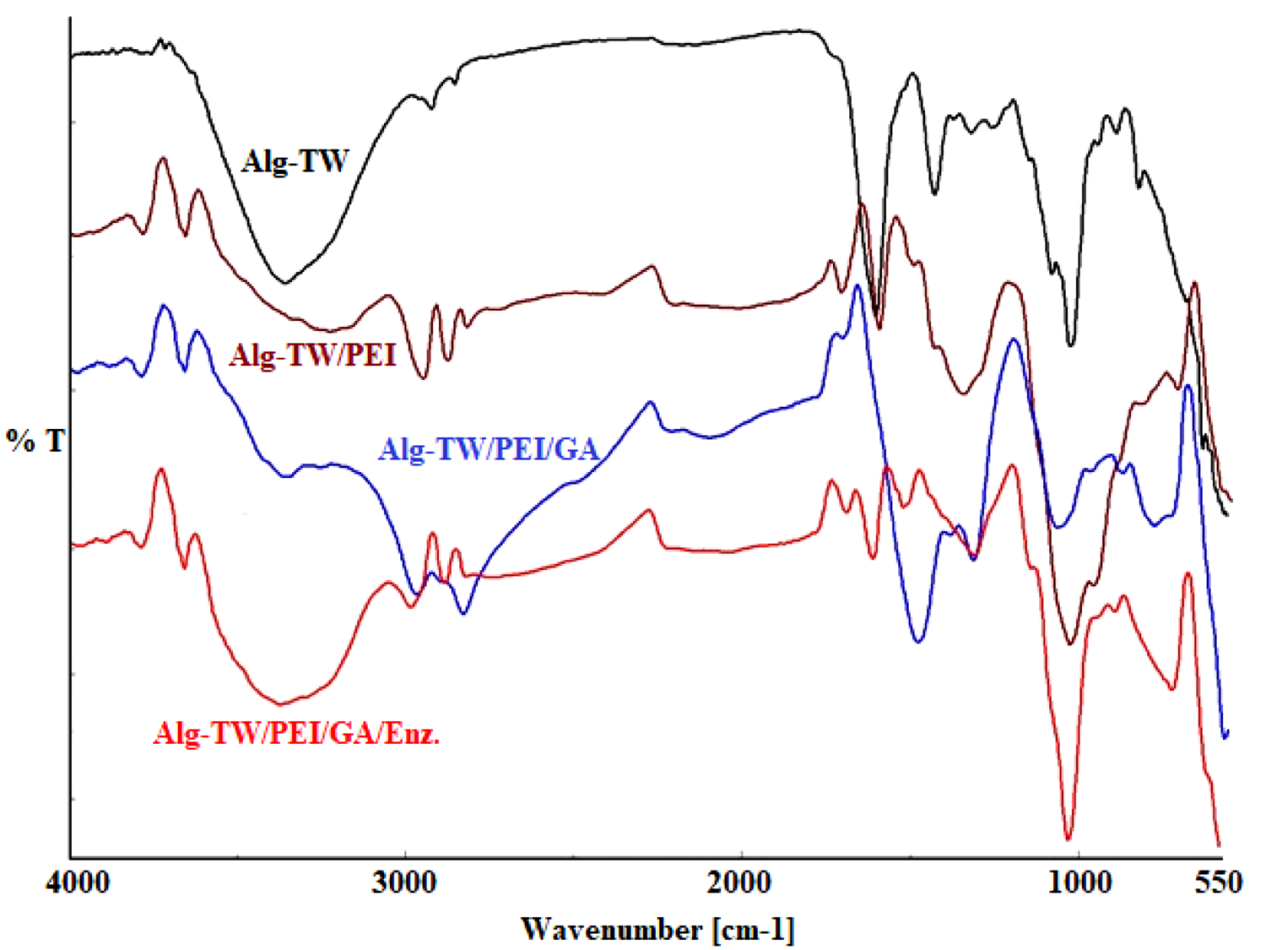
FT-IR analysis of Alg/TW gel beads (Alg/TW), Alg/TW beads treated with PEI (Alg/TW/PEI), Aminated Alg/TW beads treated with GA (Alg/TW/PEI/GA), and Activated Alg/TW beads treated with β-gal enzyme (Alg/TW/PEI/GA/Enz)

FT-IR characterizations of all formula were shown in Fig. 4, for the first formula (Alg/TW); there is a characteristic peak at 1637 cm^−1^ that corresponded to carbonyl group. Also the peak at 1632 cm^−1^ that attributed to carboxylic groups and at 3453 cm^−1^ is corresponding to (–OH) groups of tea waste. While in formula (Alg/TW/PEI); there is a new peak at 3438 cm^−1^ that of the amine groups; overlapped with peak of hydroxyl groups found on the gel beads, that amine groups produced after the reaction occurred between the Alg/TW complex and PEI. In formula (Alg/TW/PEI/GA), that contains activated gel beads with GA, the peaks at 3436 cm^−1^ is corresponding to the hydroxyl groups of GA is overlapped with that of amine groups and also hydroxyl groups that originally found on the gel beads surface. This formula also has the main characteristic peak at 1634 cm^−1^ that is corresponding to (–C=N–) bond that is formed during Schiff base interaction between amine groups found on the gel beads after amination step and the aldehyde groups of the GA. Finally for the formula (Alg/TW/PEI/GA/Enz.); it has a peak at 3436 cm^−1^ that corresponding to the amine groups of the enzyme that overlapped with the peak of amine groups and hydroxyl groups found already on the gel beads surface. Also it contain a peak at 1634 cm^−1^ that related to (–C=N–) bond that is formed during Schiff base interaction between amine groups of the enzyme and the aldehyde groups of the GA that found on the gel beads surface. These results were in agreement with the results obtained by other published results [32, 33].

### Thermal gravimetric analysis (TGA)

**Fig. 5:**
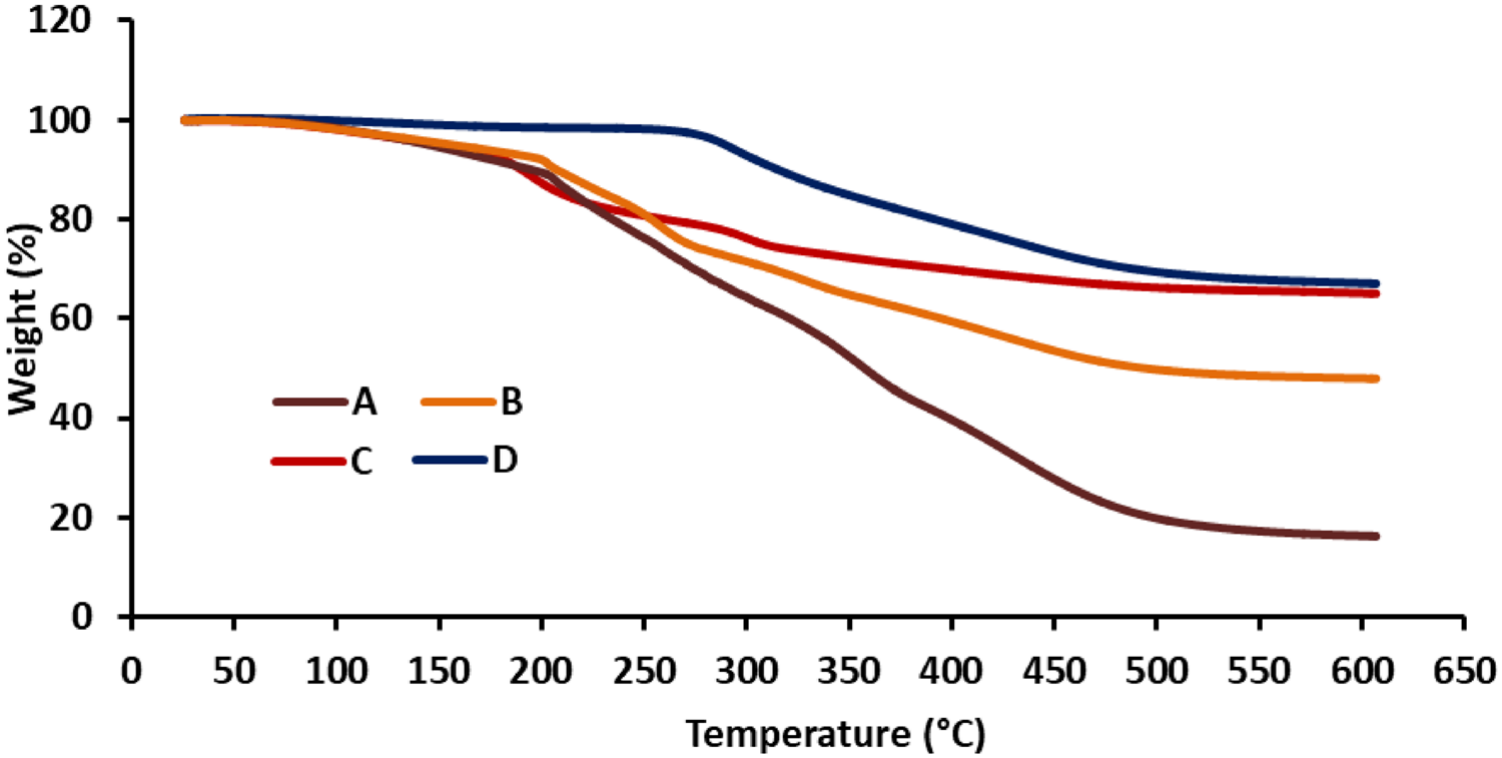
TGA of Alg/TW gel beads (**A**), Alg/TW beads treated with PEI (**B**), Aminated Alg/TW beads treated with GA (**C**), and Activated Alg/TW beads treated with β-gal enzyme (**D**)

Treatment of Alg/TW and modification by PEI and GA has shown gradual and obvious improvement in the TGA of the gel beads. The TGA of the Alg/TW and modified one (A–D), shows a better stability against thermal degradation as shown in Fig. 5. Alg/TW had degradation at 200 °C, while it was only at 163 °C in the case of alginate, as illustrated by Abeer et al. [34]**,** and at 168 °C in the case of alginate/carboxymethyle cellulose (Alg/CMC), as reported by Ghada et al. [35]. These values have been increased to become 230 °C after amination process using PEI. Also it increased to 270 °C after activation with GA and finally become 270 and 380 °C after immobilization. The gel beads thermal improvement could be explained by the formation of polyelectrolyte interaction between polyanions found in the complex and the polycations found in PEI. Further interaction with GA showed much higher increase in the thermal stability of the gel beads and the degradation behavior become slower and gradual. This improvement in TGA could be attributed to the interaction between amine groups of the PEI on the surface of the gel beads and the carbonyl group of the GA forming strong amide bond. The improvement in the thermal degradation may be due to the formation of strong bonds (Schiff's base) between the free amine groups of PEI or enzyme and the GA [34].

### Scanning electron microscopy

**Fig. 6:**
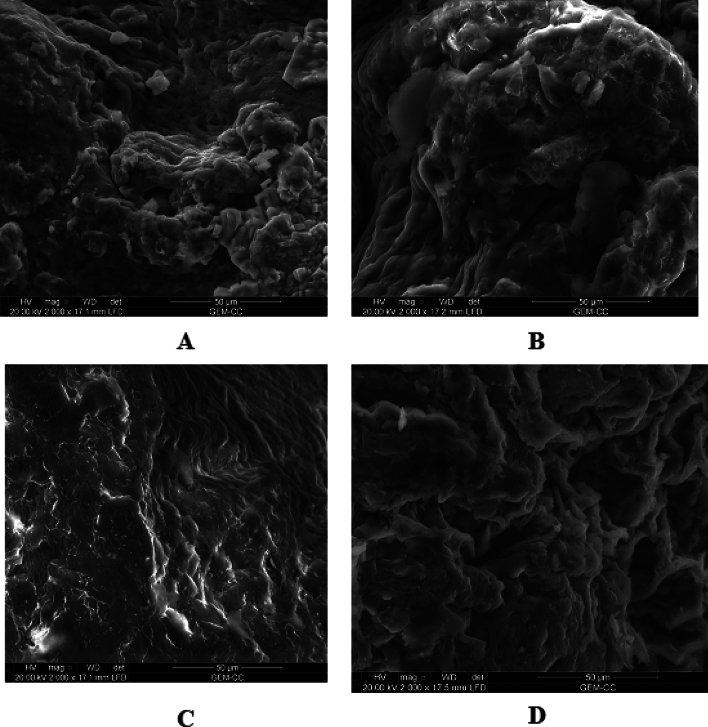
SEM of Alg/TW gel beads (**A**), Alg/TW beads treated with PEI (**B**), Aminated Alg/TW beads treated with GA (**C**), and Activated Alg/TW beads treated with β-gal enzyme (**D**)

Scanning electron microscopy analysis of four formulas (Alg/TW, Alg/TW/PEI, Alg/TW/PEI/GA, and Alg/TW/PEI/GA/Enz.) was performed to characterize the morphological structure in each step of modification and immobilization as shown in Fig. 6. As seen in Fig. 6A (Alg/TW), there are accumulations of tea waste on the surface of the gel beads that increase not only the surface area but also function groups on beads surface, also there are some pores found on the gel beads. While in Fig. 6B (Alg/TW/PEI), there are some particles on the beads surface that may be related to amine groups, and the pores become smaller. But in Fig. 6C (Alg/TW/PEI/GA), the beads surface become smoother with some wrinkles. Finally, Fig. 6D that related to immobilized one, the beads surface has some accumulations that may be related to enzyme particles [36].

### Operational stability (reusability) of immobilized β-galactosidase

**Fig. 7:**
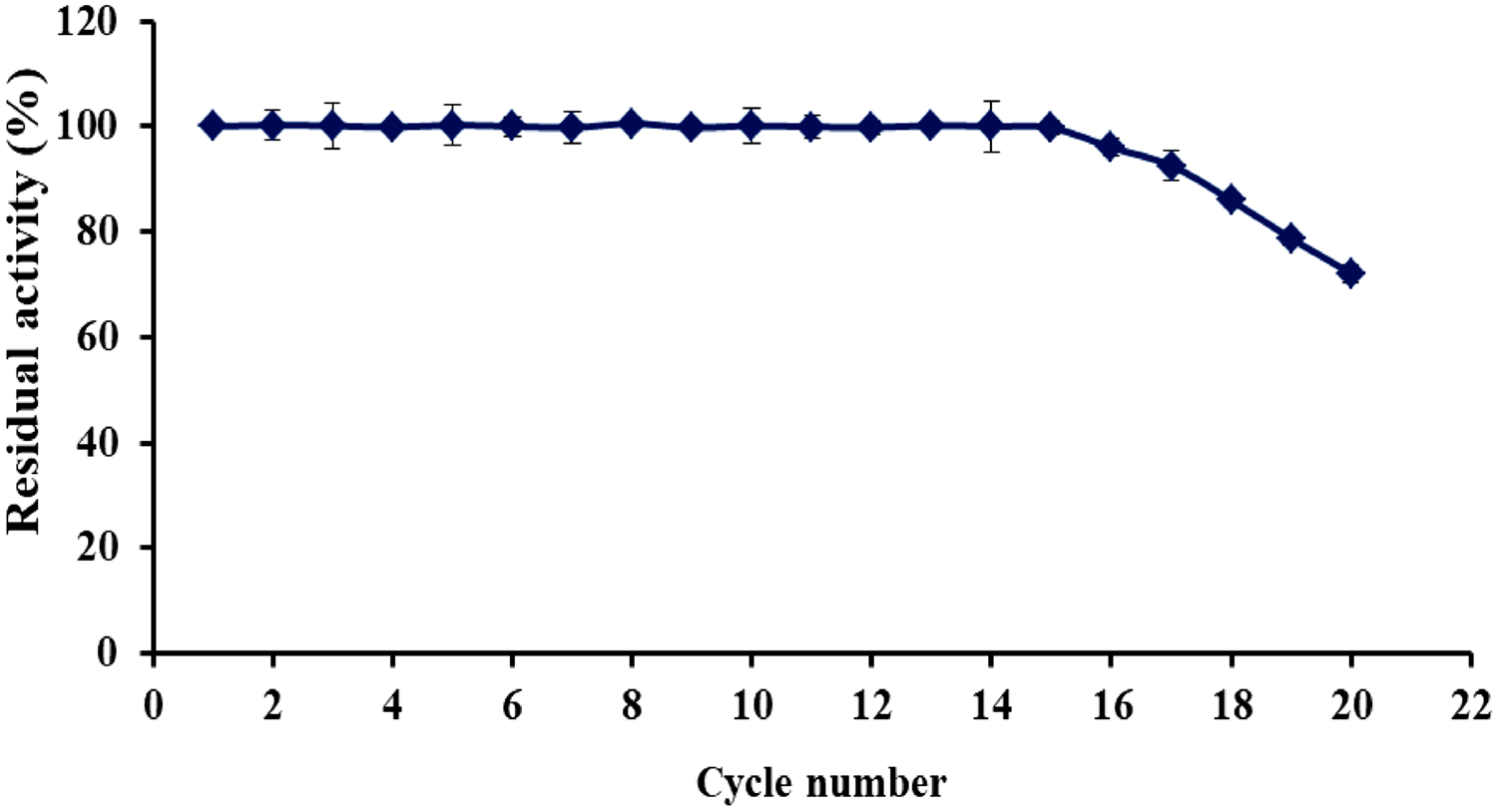
Operational stability of immobilized β-galactosidase

One of the most important advantages of immobilization process is reusability that should be considered for industrial applications due to the possibility of reusing the enzyme over a long time. The immobilization strategy of an enzyme to a support material helps in recycling it for several batches and subsequently reducing the cost of reaction system [37, 38]. The novel activated carrier Alg/TW was utilized to obtain a stable immobilized β-gal that can be separated easily from its products and restarted several reaction cycles. According to the results, the immobilized β-gal retained 99.7% of its initial activity after 15 operational cycles as shown in Fig. 7. Multipoint-covalent binding of β-gal on activated Alg/TW carrier enhanced its structural rigidity and reusability [12]. Moreover, it could be reused for 20 consecutive cycles conserving 72.1% of its original activity. This implies the immobilized β-gal on Alg–TW/PEI/GA carrier had an excellent durability, recovery, and operational stability. Our finding is superior to that given by Ansari et al. [25] who suggested that, the reusability of immobilized β-gal exhibited 89% residual activity after consecutive 5 cycles. On the other hand, the immobilized β-gal retained more than 90% of its initial activity even after 15 runs as reported by Chen et al. [39].

When we compare the reusability of the immobilized enzymes on alginate modifications, we notice that the immobilized laccase enzyme lost about 40% of its activity after 10 cycles when immobilized on alginate beads [34], and the immobilized inulinase enzyme lost more than 50% of its activity after 12 cycles when immobilized on alginate-CMC beads [35]. In addition, the residual activity of immobilized β-gal after 12 cycles was approximately 51% when immobilized on a UV-cured epoxy-based polymeric film [40]. The cross-linked β-gal maintained 88.02% activity even after the third cycle when immobilized on ZnO nanoparticles, as investigated by Selvarajan et al. [41]. Moreover, the immobilized β-gal kept 83% of its initial activity after sixth repeated use, as described by Alshanberi et al. [42].

## Conclusion

β-galactosidase was covalently immobilized on Alg/TW carrier that was chemically modified with PEI followed by GA. Chemical modification parameters of Alg/TW beads were statistically optimized by Response Surface methodology (RSM) using Box–Behnken design (BBD). The ANOVA results including *F* value (37.26), *P* value, *R*^2^ value (0.9882), Adjusted *R*^2^ value (0.9617) and predicted *R*^2^ value (0.8130) confirmed the good fitness and significance of BBD. In addition, the adequate precision and reliability of the experimental results were achieved from the low CV value (2.90) of the statistical model. The highest IY % (75.1%) of β-gal using BBD was obtained at PEI concentration (4%), PEI pH (9.5), and GA concentration (2.5%). In addition, FT-IR, TGA, and SEM analysis were employed to investigate all stages of enzyme immobilization process. Finally, the immobilized β-gal retained 99.7% and 72.1% of its initial activity after 15 and 20 consecutive cycles.

## Data Availability

All data generated or analysed during this study are included in this published article.
